# RNA-binding proteins Zfp36l1 and Zfp36l2 protect against premature thymic involution

**DOI:** 10.1038/s41423-026-01399-7

**Published:** 2026-03-16

**Authors:** Jianxun Han, Mahdieh Golzari-Sorkheh, Vinothkumar Rajan, Juan Carlos Zúñiga-Pflücker

**Affiliations:** 1 Biological Sciences, Sunnybrook Research Institute, Toronto, ON Canada; 2 Department of Immunology, University of Toronto, Toronto, ON Canada

**Keywords:** Thymus epithelial cells, Foxn1, AU-rich elements, Central tolerance, Inflammatory cytokines, T cells, Thymus

## Abstract

The thymus is a primary lymphoid organ in which diverse and self-tolerant T cells are produced from bone marrow-derived hematopoietic progenitors. Progressive, age-associated thymic involution reduces T-cell output and impairs adaptive immunity; however, the molecular mechanisms underlying this process remain elusive. Here, we report that the conditional deletion of the RNA-binding proteins Zfp36l1 and Zfp36l2 in thymic epithelial cells (TECs) leads to a pronounced reduction in the number of TECs during the embryonic stage and early neonatal stage, despite a largely preserved thymus size. Postnatally, these mice exhibit excessive medullary TEC (mTEC) expansion, elevated intrathymic proinflammatory cytokine production, FOXN1 downregulation, and premature thymic involution. These findings reveal a protective role for Zfp36 Tristetraprolin (TTP) family proteins in regulating cytokine levels within the thymic microenvironment and preventing premature thymic involution. Moreover, our results suggest a previously unappreciated connection between central tolerance induction and the onset of age-associated thymic involution.

## Introduction

The thymus is a primary lymphoid organ essential for the development of a diverse and self-tolerant T-cell repertoire. Thymic epithelial cells (TECs) are broadly classified into cortical TECs (cTECs) and medullary TECs (mTECs) on the basis of their location. cTECs provide crucial signals that regulate the lineage commitment, migration, differentiation, proliferation, and survival of developing thymocytes during the early stages of T-lymphopoiesis, whereas mTECs play a central role in establishing central tolerance at later stages by expressing a broad array of tissue-restricted antigens (TRAs) [1]. The expression of TRAs is regulated in a complementary manner by the transcription factors AIRE and FEZF2 [2, 3]. Mutations in *AIRE* have been directly linked to the development of autoimmune disorders [4, 5**]**.

The thymus undergoes progressive age-related involution, as well as acute stress-induced atrophy, resulting in diminished T-cell output and compromised adaptive immunity [6, 7]. The downregulation of Forkhead box N1 (Foxn1), a master transcription factor critical for TEC development, maintenance, and survival, has been implicated as a key driver of age-associated thymic involution [8, 9]. Conversely, forced expression of *Foxn1* in aged TECs has been shown to partially reverse thymic atrophy and restore their T-lymphopoietic capacity [10], underscoring the central role of Foxn1 in sustaining thymic function throughout life.

Chronic inflammation, marked by persistent elevation of proinflammatory cytokines, is increasingly recognized as a central driver of diverse pathologies, including autoimmune disorders, cardiovascular disease, and cancer [11]. Several inflammatory mediators, most notably type I interferons (IFNs), interleukin (IL)-6, and tumor necrosis factor (TNF)α, have been directly implicated in both acute thymic atrophy and age-associated thymic involution. Notably, gene targeting (knockout) of the type I IFNα receptor (*Ifnar1*) or treatment with a neutralizing antibody against IFNAR1 significantly mitigates acute thymic atrophy induced by chronic lymphocytic choriomeningitis virus (LCMV) infection or poly(I: C) treatment [12**–**13]. In the context of poly(I: C) treatment, the protective effect appears to be mediated through the direct action of IFNs on thymic stromal cells, as conditional *Ifnar1* knockout in T and B cells fails to prevent thymic atrophy [12]. Consistent with this mechanism, recombinant human type I IFN treatment induces differentiation, growth arrest, and apoptosis in a human cTEC cell line [14]. Similarly, IL-6 expression is elevated in the aged human thymus, and intraperitoneal injection of IL-6 in young mice leads to acute thymic atrophy [15]. Finally, the Nlrp3 inflammasome, which is regulated by TNFα, has been shown to promote age-associated thymic involution, and its genetic ablation attenuated this process and enhanced T-cell output in old mice [16]. Notably, mTECs express a wide array of cytokines whose potential functions have not been fully investigated [17].

The tristetraprolin (TTP) family, primarily comprising TTP/Zfp36, Zfp36l1, and Zfp36l2, is a group of RNA-binding proteins that bind to AU-rich elements (AREs) within the 3’-untranslated regions (3’-UTRs) of target mRNAs to promote their degradation. Through this posttranscriptional regulation, TTP family members exert broad control over genes encoding oncogenic or tumor-suppressive transcription factors, cell cycle and survival regulators, and cytokines and have been strongly implicated in the pathogenesis of cancer and autoimmune disorders [18, 19]. Consistent with this role, Zfp36-deficient mice develop a complex autoimmune syndrome driven by excessive TNFα production [20]. Similarly, the conditional deletion of Zfp36l1 and Zfp36l2 in early developing thymocytes results in impaired T-lymphopoiesis and the emergence of T-cell acute lymphoblastic leukemia (T-ALL) as a consequence of stabilization of *Notch1* mRNA [21].

Despite their well-established roles in hematopoietic cells, the functions of TTP family members in TECs have not been investigated. To address this gap, we created conditional Zfp36l1 and Zfp36l2 double knockout mice using a *Foxn1-cre* driver for TEC-specific deletion to explore their functions, particularly in relation to thymic involution. Our findings reveal that Zfp36l1 and Zfp36l2 function redundantly within TECs to regulate intrathymic inflammatory cytokine levels, thereby preventing premature thymic involution. Collectively, these results suggest that physiological levels of inflammatory cytokines may contribute to progressive, age-associated thymic involution and highlight the essential role of TTP family members in fine-tuning the balance between central tolerance induction and protection against premature thymic involution.

## Results

### Zfp36l1 and Zfp36l2 in TECs redundantly regulate thymus cellularity

**Fig. 1:**
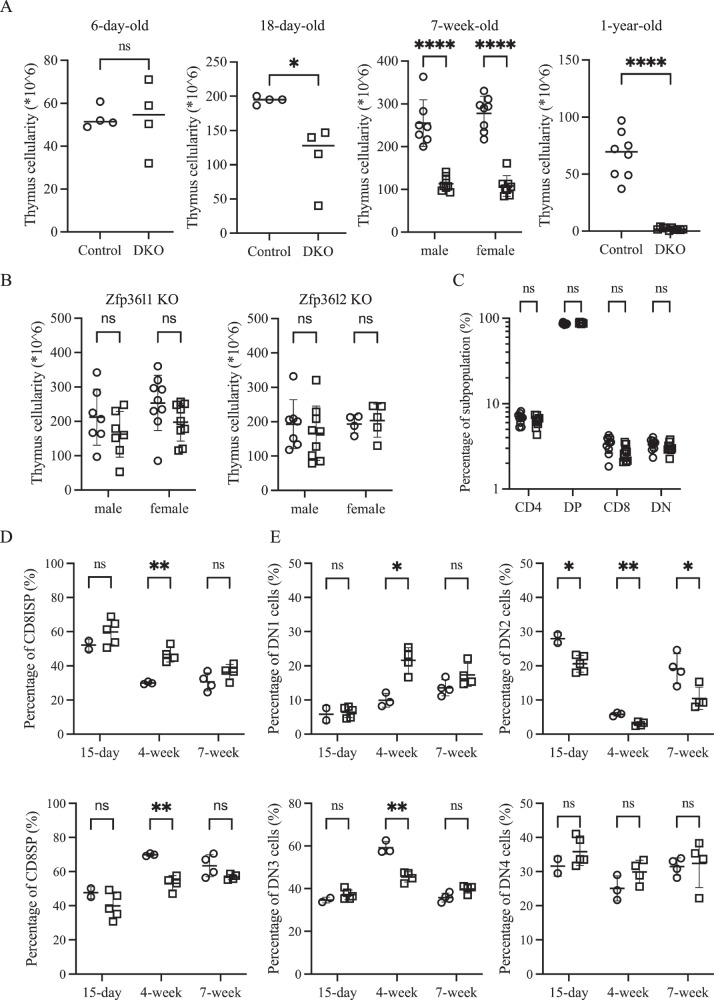
Zfp36l1 and Zfp36l2 in TECs redundantly regulate thymus size.** A** Total thymus cellularity is compared between floxed control (○) and DKO (□) mice at four different ages, revealing a progressive decrease in DKO mice. **B** Scatter plots showing thymus cellularity in 7-week-old control mice (○) versus mice deficient in *Zfp36l1* (left) or *Zfp36l2* (right) (□), highlighting the dominant role of Zfp36l1. **C** Frequencies of four major thymocyte subsets (based on CD4 and CD8 expression) were compared between 7-week-old control (○) and DKO (□) mice. **D** The percentages of CD8ISP (top) and CD8SP among CD4^-^CD8^+^ thymocytes at three different ages are skewed toward the CD8ISP in DKO mice (□) compared with those in controls (○) at 4 weeks of age. **E** Scatter plots comparing the frequency of four DN subsets between control (○) and DKO mice (□) at three different ages, with a notable reduction in DN2 and DN3 cells in DKO mice

To investigate whether the stabilization of *Dll4* mRNA, which has a short half-life because of the presence of AREs within its 3’-UTR (unpublished observation), could delay age-associated thymic involution, we generated a conditional double knockout (DKO) mouse strain in which two ARE binding proteins, Zfp36l1 and Zfp36l2, were specifically deleted in TECs using a *Foxn1-cre* driver. Contrary to our expectation, one-year-old DKO mice exhibited markedly reduced thymus cellularity, which decreased to less than 10% of that observed in floxed controls (Fig. 1A). Analysis of younger DKO mice revealed that thymus hypoplasia was already evident at three weeks of age **(**Fig. 1A**)**. Examination of 7-week-old single conditional knockout mice revealed that Zfp36l1 plays a dominant role in TECs, whereas Zfp36l2 provides incomplete compensation. While Zfp36l2 deficiency alone did not affect thymic cellularity, Zfp36l1 single-knockout mice exhibited a consistent trend toward decreased cellularity, although the difference did not reach statistical significance (Fig. 1B).

Despite this profound decline in cellularity, the relative distribution of the 4 major thymocyte subsets, defined by CD4 and CD8 expression, remained largely preserved (Fig. 1C and Supplementary Fig. 1 for gating strategies). However, longitudinal analyses revealed more subtle developmental perturbations. Specifically, in 4-week-old DKO mice, the ratio of CD8 immature single-positive (ISP) cells to mature CD8 single-positive (SP) cells was skewed toward CD8ISP cells (Fig. 1D). Additionally, within the CD4^-^CD8^-^ double-negative (DN) subset, DKO thymuses exhibited relatively fewer DN2 and DN3 cells and a greater proportion of DN1 cells, while the DN4 cell frequency was comparable to that of the controls (Fig. 1E). With the exception of a persistent reduction in DN2 cells, these differences were not observed at either 15 days or 7 weeks of age.

### Loss of Zfp36l1/2 in TECs alters the mTEC phenotype in adult mice

**Fig. 2:**
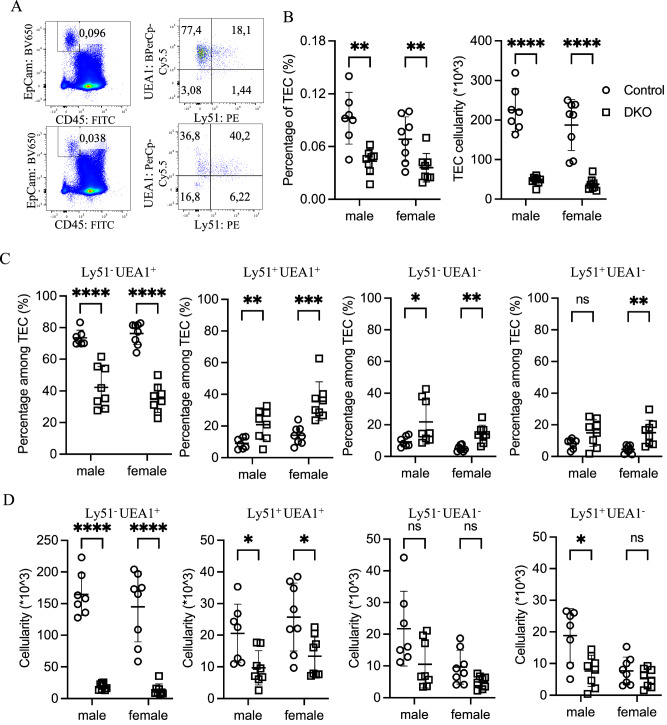
Zfp36l1 and Zfp36l2 deficiency in TECs leads to a severe reduction in mTECs in adult mice.** A** Representative flow cytometry pseudocolor plots display the percentages of total TECs and their subpopulations in 7-week-old control (top) and DKO (bottom) mice. **B** Scatter plots comparing the percentage (left) and absolute number (right) of total TECs between 7-week-old control (○) and DKO (□) mice. **C** Percentages of four TEC subpopulations are shown for control (○) and DKO (□) mice, revealing an altered mTEC profile in DKO mice. **D** Absolute numbers of TEC subpopulations were compared between control (○) and DKO (□) mice, and a pronounced reduction in mTECs was detected in DKO mice

To further characterize the potential effects of the loss of Zfp36l1/2 on TECs, we analyzed TECs that were enzymatically dissociated from seven-week-old thymuses by flow cytometry. TECs, identified as EpCAM+ CD45^-^ cells, were reduced in frequency among total thymic cells by approximately 50% in DKO mice compared with those in controls (Fig. 2A, B). Combined with the decrease in total thymocyte numbers, this led to an ~80% reduction in the number of recovered TECs, with control mice having ~2 × 10^5^ TECs and DKO mice having ~4 × 10^4^ TECs (Fig. 2B).

Consistent with previous reports that enzymatic preparation preferentially damages cTECs relative to mTECs [22, 23], most recovered TECs were UEA1^+^Ly51^–^ mTECs (Fig. 2C, D). However, DKO mTECs exhibited an altered surface marker profile, with many becoming atypical UEA1^+^Ly51^+^ or UEA1^–^Ly51^–^ cells, which were less common in control thymuses (Fig. 2C, D). While the increased frequency of UEA1^–^Ly51^–^ cells likely reflects an expansion of the recently reported age-associated TEC population [24], the accumulation of UEA1^+^Ly51^+^ cells may indicate developmental arrest en route to either UEA1^+^Ly51^–^ mTECs or UEA1^-^Ly51^+^ cTECs. Alternatively, UEA1^+^Ly51^+^ cells may represent mature mTECs expressing Ly51. Additionally, a significant increase in the proportion of UEA1^–^Ly51^+^ cTECs was observed in the thymus of DKO mice (Fig. 2C). Analysis of single-gene knockouts supported the dominant role of Zfp36l1 over Zfp36l2 in TECs (Supplementary Fig. 2).

### Preferential loss of cTECs in the neonatal DKO thymus

**Fig. 3:**
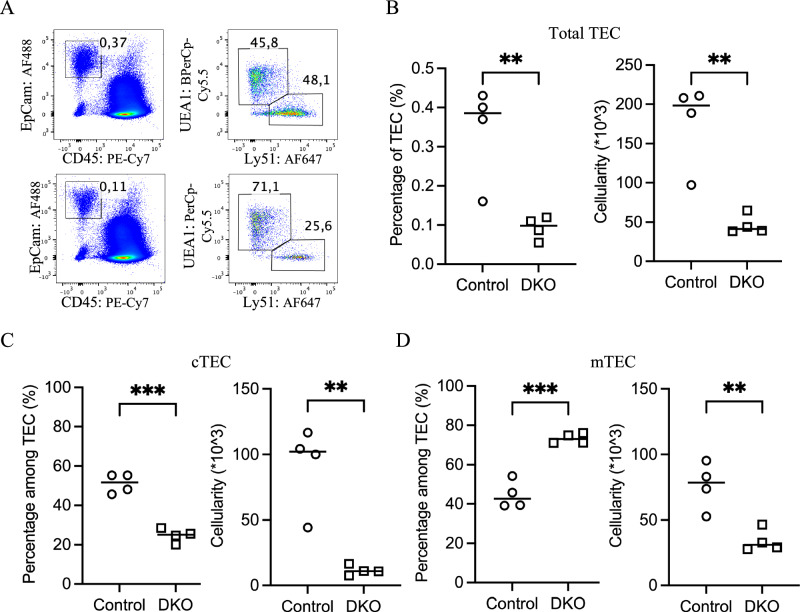
Preferential loss of cTECs in the neonatal DKO thymus**. A** Representative flow cytometry pseudocolor plots illustrate the percentages of total TECs and their subpopulations in 6-day-old control (top) and DKO (bottom) thymuses. **B** Scatter plots comparing the percentage (left) and absolute number (right) of total TECs between control (○) and DKO (□) mice at postnatal day 6. **C** The percentage (left) and absolute number (right) of Ly51^+^UEA1^-^ cTECs were compared between control (○) and DKO (□) mice, and a marked reduction was detected in DKO mice. **D** The percentage (left) and absolute number (right) of Ly51^-^UEA1^+^ mTECs were compared between control (○) and DKO (□) mice, with DKO mice increasing in number but decreasing in absolute number

To determine the developmental stage at which TEC loss initiates, we examined neonatal mice at postnatal day 6 (P6), when total thymic cellularity was comparable between DKO and control mice (Fig. 1A). Nonetheless, compared with that in control mice, the frequency of TECs among total thymic cells in DKO mice was decreased by approximately 75% (Fig. 3A, B), corresponding to a substantial reduction in absolute TEC numbers. Analysis of TEC subsets revealed that UEA1^–^Ly51^+^ cTECs, which accounted for nearly half of the TECs in control mice, were markedly reduced in DKO mice, constituting less than 30% of the total TEC population (Fig. 3C). As a result, the proportion of UEA1^+^Ly51^–^ mTECs increased, although their absolute cell numbers were lower than those in the controls (Fig. 3D). These results indicate that TEC loss initiates before birth, with cTEC showing a earlier reduction than mTEC.

### Single-cell RNA sequencing (scRNA-seq) reveals abnormal expression levels of cell cycle- and cell fate-related genes in DKO TECs

To elucidate the molecular mechanisms underlying the observed TEC defects in Zfp36l1/2 DKO mice, we performed scRNA-seq on TECs sorted from neonatal thymuses (P6), which permitted us to investigate the effects of Zfp36l1 and Zfp36l2 deficiency on global gene expression in both the cTEC and mTEC subsets.

**Fig. 4:**
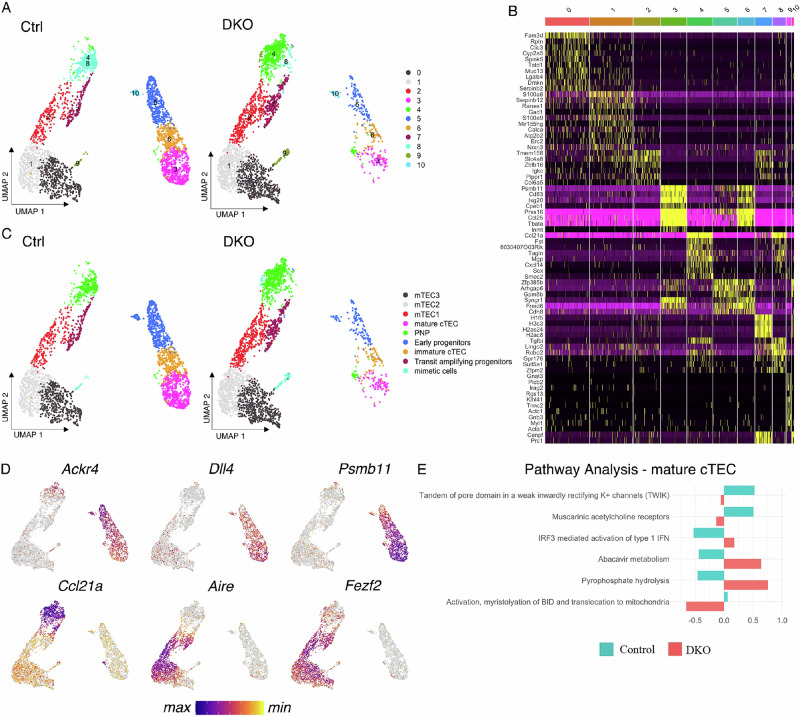
TECs from 6-day-old control and DKO thymuses exhibit distinct gene expression profiles. **A** UMAP plots visualizing TEC subpopulations based on transcriptomic profiles from 6-day-old control and DKO thymuses. **B** A heatmap displays signature genes defining individual TEC clusters. **C** Cluster annotation based on signature genes. **D** Feature plots illustrating the expression patterns of six representative TEC genes, *Ackr4*, *Dll4*, *Psmb11*, *Ccl21a*, *Fezf2*, and *Aire*. **E** Pathway analysis revealed enrichment of the type I IFN signaling pathway in DKO mature cTECs compared with controls

Unsupervised clustering revealed two distinct groups corresponding to cTECs and mTECs, with more subclusters within each group (Fig. 4A). Gene signatures of individual clusters revealed a clear differentiation and maturation trajectory extending from the top to the bottom of each island, with the respective progenitor cells—cluster 5 for cTECs and clusters 4 and 8 for mTECs—positioned at the top (Fig. 4B). Notably, two of the six signature genes in cluster 5, *Frmd6* and *Gpm6b*, were among the previously identified gene signatures for cTEC-biased early progenitors [25]. Additionally, *Ackr4*, another signature gene of early progenitors identified in a previous study, exhibited its highest expression in cluster 5 (Fig. 4D). Similarly, six of the fourteen genes that were highly expressed in clusters 4 and 8, namely, *Ccl21a, Fst, Mgp, Tagln, Tgfbi*, and *Sult5a1*, corresponded to signature genes associated with mTEC-biased postnatal progenitor cells [25]. In particular, the strong expression of *Ccl21a* in clusters 4 and 8 aligns with recent findings that the majority of adult mTECs are derived from *Ccl21a*-expressing cells [26]. In further support of the inferred maturation trajectory, a gradual increase in the expression of *Dll4* and *Psmb11* was observed from the top to the bottom of the cTEC island, whereas the spatial expression patterns of *Fezf2* and *Aire* across the mTEC island were consistent with progressive differentiation (Fig. 4D). On the basis of these transcriptional profiles, the clusters were annotated as follows: early progenitors (cluster 5), immature cTECs (cluster 6), mature cTECs (cluster 3), postnatal progenitors (PNP, clusters 4 and 8), mTEC1 (cluster 2), mTEC2 (cluster 1), mTEC3 (cluster 0), and transit-amplifying progenitors (cluster 7) (Fig. 4C). Transit-amplifying progenitors are characterized by elevated expression of histones, *Cenpf* and *Prc1*, two genes with known functions in cell cycle progression, particularly mitosis.

Consistent with the flow cytometry data, neonatal DKO TECs exhibited a pronounced transcriptional bias toward the mTEC lineage, with most cells localized within clusters 0, 1, 2, 4, 7, and 8. The distribution of cells between control and DKO samples differed significantly across all clusters (Supplementary Fig. 3A). RNA velocity analysis further revealed that postnatal progenitors in DKO mice appeared to be more developmentally immature, as they were predominantly located in cluster 4, whereas their control counterparts were primarily found in cluster 8 (Supplementary Fig. 3B). Additionally, most DKO cTECs were positioned at the periphery of clusters 3, 5, and 6, supporting their immature phenotype.

Among the differentially expressed genes, there were numerous transcription factors and several cell cycle–related genes (Supplementary Table 1). Notably, the expression of *Tshz2*, a putative tumor suppressor that represses cell cycle progression when overexpressed in a normal mammary cell line [27], increased across most clusters in DKO TECs compared with controls. Although the functional impact of its increased expression in TECs remains to be determined, it is plausible that it interferes with cell cycle progression through a mechanism similar to that in mammary cells. Furthermore, the expression of *Cdkn1a* and *Cdkn1c*, which encode the cell cycle inhibitors p21 and p57, respectively, was upregulated in multiple clusters of DKO TECs compared with control cells. Specifically, *Cdkn1a* expression was elevated in early progenitors, immature cTECs, mature cTECs, and postnatal progenitors, whereas *Cdkn1c* expression was increased in postnatal progenitors, mTEC1, and transit-amplifying progenitors. Together, their upregulation likely slowed cell cycle progression and consequently led to fewer TECs in the thymus of DKO mice.

### Higher levels but lower frequencies of FOXN1 expression in embryonic DKO TECs

**Fig. 5:**
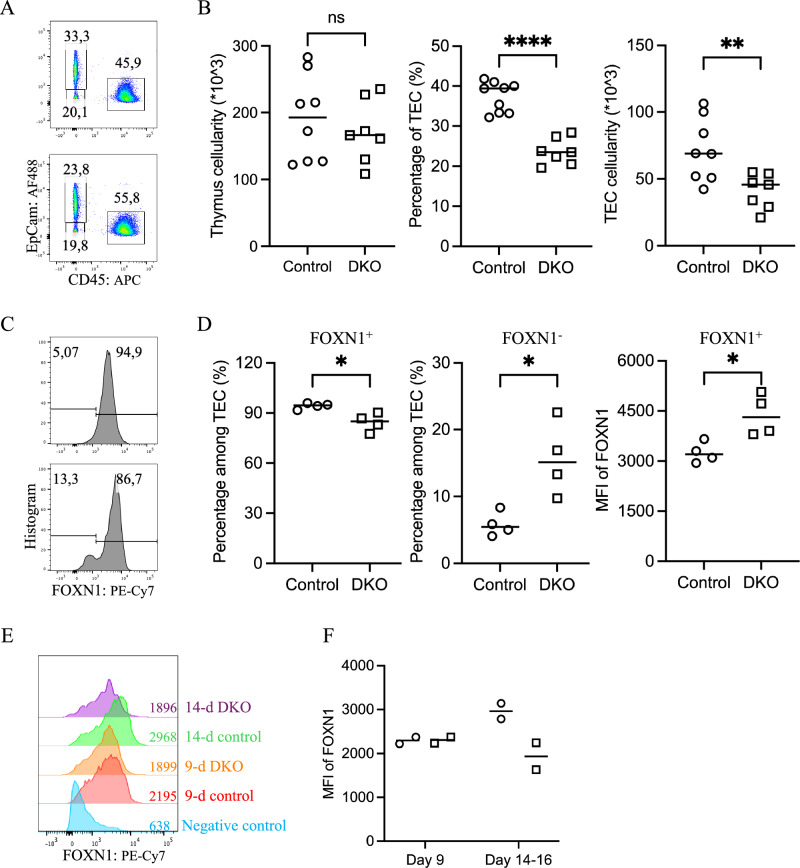
Increased FOXN1 levels but reduced FOXN1+ TEC frequency in embryonic DKO thymuses. **A** Representative flow cytometry pseudocolor plots comparing major thymic cell populations (CD45^+^ thymocytes, CD45^-^EpCam^+^ TECs, and CD45^-^EpCam^-^ non-TECs) between control (top) and DKO (bottom) E15.5 thymuses. **B** Scatter plots showing total thymic cellularity (left), percentage (middle), and absolute number (right) of TECs between control (○) and DKO (□) E15.5 thymuses. **C** Representative flow cytometry histograms comparing the percentages of FOXN1-expressing TECs and FOXN1 levels in control (○) and DKO (□) E15.5 thymuses. **D** Scatter plots comparing the frequencies of FOXN1^+^ TECs (left), FOXN1^-^ TECs (middle), and the mean fluorescence intensity (MFI) of FOXN1^+^ cells (right) between control (○) and DKO (□) thymuses at E15.5, revealing a decreased frequency of FOXN1-expressing cells but higher FOXN1 levels in DKO thymuses. **E** Overlay of flow cytometry histograms showing FOXN1 protein levels in cTECs from 9-day-old and 14-day-old mice. The mean fluorescence intensity (MFI) values are indicated. **F** A scatter plot of the MFI values of the FOXN1 protein in cTECs

The elevated expression of negative cell cycle regulators in DKO TECs, along with the observed reduction in TEC cellularity in the neonatal thymus, prompted us to investigate whether the decreased TEC numbers were a result of impaired proliferation during embryonic thymus development. To assess this, we performed EdU pulse-labeling in timed-pregnant mice. However, no significant differences in the percentage of EdU-positive TECs, which represent cells in the S and early G2 phases, were detected between the DKO and control groups at any of the three embryonic time points analyzed: E13.5, E15.5, and E18.5 (Supplementary Fig. 4A, B). Nonetheless, a reduction in the percentage of TECs in DKO thymuses compared with that in controls was evident as early as E15.5 (Figs. 5A, B and Supplementary Fig. 4C). This phenotype mirrors that seen in the iFoxn1 mouse strain, which expresses ectopic *Foxn1* in TECs (Supplementary Fig. 4C). Given that *Foxn1* mRNA contains an ARE within its 3’UTR (Supplementary Fig. 4D) and that it is a potential direct target of Zfp36l1/2-mediated mRNA decay, we assessed FOXN1 protein levels in E15.5 embryonic TECs by intracellular flow cytometry. Among FOXN1-expressing TECs, DKO cells presented significantly higher FOXN1 protein levels than control cells did (Figs. 5C, D and Supplementary Fig. 4E). Conversely, a greater proportion of DKO TECs lacked detectable FOXN1 expression. The elevated FOXN1 protein levels within the expressing TEC population and the consequent upregulation of the expression of prolymphopoietic FOXN1 target genes such as *Dll4*, *Ccl25*, and *Cxcl12* may compensate for the reduced number of TECs in supporting thymocyte development during the embryonic and neonatal stages. However, despite elevated FOXN1 protein levels in DKO TECs during fetal development, FOXN1 expression decreased to levels below those observed in control mice by two weeks postnatally (Fig. 5F and Supplementary Fig. 4F).

### Elevated levels of inflammatory cytokines contribute to premature thymic involution

**Fig. 6:**
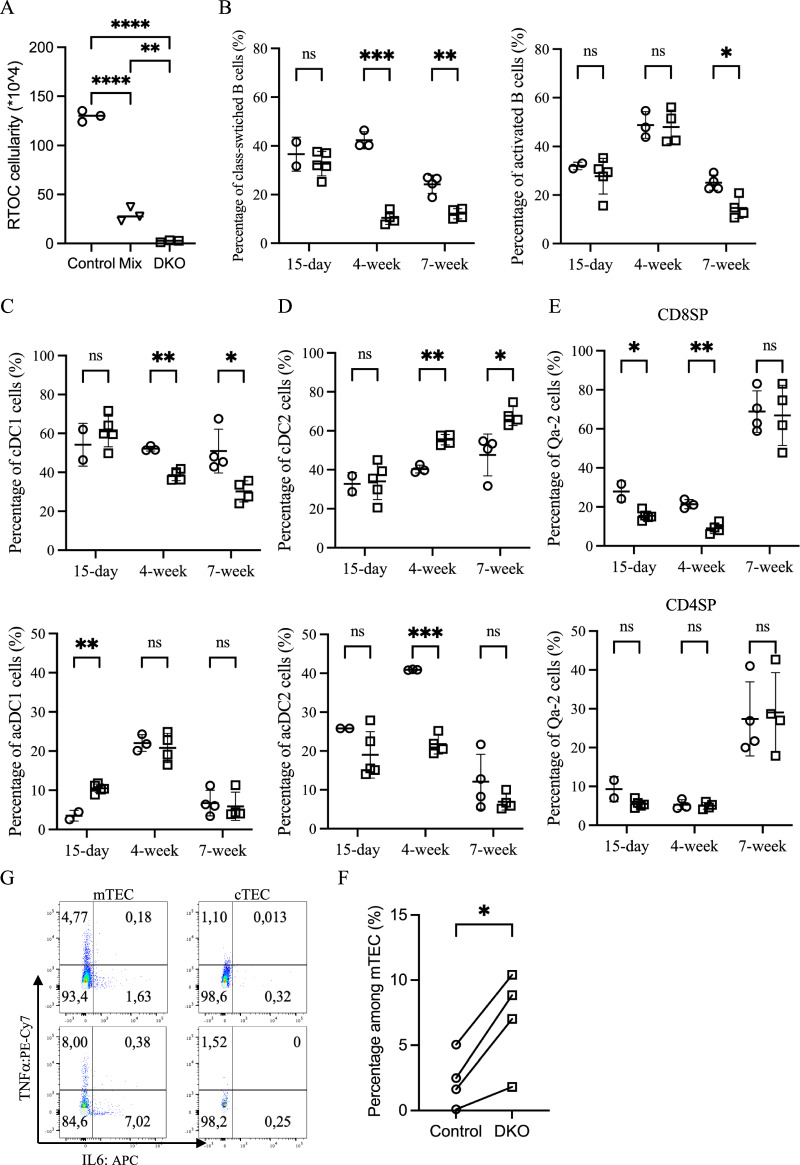
Elevated inflammatory cytokine levels contribute to early thymic involution in DKO mice**. A** A scatter plot comparing total cell numbers after 3 weeks of RTOC culture using stromal cells from control (○), DKO (□), or a 1:1 mixture of control and DKO stromal cells (∇); statistical significance was assessed by one-way ANOVA. **B** Scatter plots showing the percentages of class-switched B cells as defined by B220^+^CD11c^-^IgD^-^Ly6c^+^ (left) and activated B cells as defined by B220^+^CD11c^-^IgD^-^GL7^+^ (right) in control (○) and DKO mice (□) at three different ages, with DKO thymic B cells displaying a less active phenotype. **C** Percentages of cDC1 cells as defined by B220^-^CD11c^+^MHCII^+^SIRPα^-^XCR1^+^ (top) and activated cDC1 (acDC1) cells as defined by MHCII^high^CCR7^+^ cDC1 cells (bottom) in control (○) and DKO mice (□) at three different ages. **D** Percentages of cDC2 cells as defined by B220^-^CD11c^+^MHCII^+^SIRPα^+^XCR1^-^ (top) and activated cDC2 (acDC2) cells as defined by MHCII^high^CCR7^+^ cDC2 cells (bottom) in control (○) and DKO mice (□) at three different ages. **E** Percentages of very late-stage mature single-positive (SP) cells as defined by CD3^+^TCRβc^+^CD69^-^CD24^low/-^MHCI^+^Qa-2^+^ with CD8SP (top) and CD4SP (bottom) in control (○) and DKO mice (□) at three different ages. **G** Flow cytometry pseudocolor plots comparing IL6 and TNFa expression in mTECs and cTECs after 4 h of GolgiStop treatment in 9-day-old control (top) and DKO (bottom) mice. **F** A scatter plot with a paired *t* test was constructed to compare the percentages of IL6^+^ mTECs after 4 h of GolgiStop treatment in control (○) and DKO (□) mice from postnatal day 9 to 3 weeks of age, revealing increased IL6 expression in DKO mTECs

To investigate whether the notable early thymic involution in DKO mice results from an autonomous deleterious effect of Zfp36l1 and Zfp36l2 deficiency in TECs, we compared the growth of reaggregated thymus organ cultures (RTOCs) composed of stromal cells from (1) floxed control thymus only, (2) Zfp36l1/2-deficient thymus only, or (3) an equal mixture of both genotypes. Although all RTOCs developed normally during the first week (Supplementary Fig. 5A), by the end of the three-week culture period, both RTOCs derived solely from DKO stroma and, more importantly, the mixed chimeric stroma had collapsed, indicating a cell-autonomous deleterious effect on DKO TECs (Fig. 6A). This observation prompted further investigation into the molecular mechanisms underlying this TEC-intrinsic dysfunction.

Among the genes regulated by the TTP family of RNA-binding proteins, cytokines, including proinflammatory cytokines, represent a well-established class, in addition to transcription factors and cell cycle regulators [18]. mTECs produce type I IFNs [28], and our signaling pathway analysis of the scRNA-seq data suggested that genes related to type I IFN activation were enriched in DKO mature cTECs compared with their control counterparts (Fig. 4E). Both our group and others have shown that type I IFNs contribute to thymic atrophy during chronic LCMV infection and poly(I: C) treatment [13, 29]. On the basis of these findings, we examined whether potentially elevated type I IFN levels contributed to early thymic involution. However, transplantation of type I IFN-resistant *Ifnar1*-deficient BM did not lead to significant restoration of thymic cellularity in DKO mice (Supplementary Fig. 5B).

Recent studies have shown that intrathymic IFNs activate and license antigen-presenting dendritic cells (DCs) and B cells [30, 31]. Therefore, we examined the activation profiles of thymic DCs and B cells, as well as their impact on thymocyte maturation. Compared with control mice, thymic B cells from adolescent (4–7 weeks), but not infant (postnatal day 15), DKO mice exhibited lower licensing and activation profiles, as indicated by lower frequencies of Ly6c^+^ or GL7^+^ cells among IgD^-^ cells (Fig. 6B). In parallel, adolescent DKO mice exhibited an altered thymic DC composition, with a relative increase in the ratio of cDC2 to cDC1 subsets (Fig. 6C, D). Notably, fewer cDC2s upregulated the expression of the activation marker CCR7 at 4 weeks of age, a difference that was no longer apparent at 7 weeks. Conversely, cDC1s from the infant DKO thymus showed increased activation, an effect that was lost in adolescent mice. Consistent with these changes, the maturation of CD8SP thymocytes was impaired in infant and early adolescent DKO mice, as evidenced by reduced frequencies of late-stage mature CD69^-^CD24^low/-^MHCI^hi^Qa-2^+^ cells, which was consistent with a decreased proportion of CD8 T cells in the spleen (Fig. 6E and Supplementary Fig. 5C). In contrast, CD4SP maturation appeared to be unaffected. Although these findings contrast with the expectation that elevated IFN levels uniformly increase DC and B-cell activation, they instead suggest a reshaped thymic microenvironment in which additional inflammatory cues may modulate antigen-presenting cell function.

To explore the possibility of altered cytokine expression levels, neonatal TECs from DKO and control mice were treated with GolgiStop for 4 hours, followed by intracellular staining for IL-6 and TNFα. Compared with control mTECs, DKO mTECs expressed significantly more IL-6 and TNFα, although only the increase in IL-6 was statistically significant. In contrast, cTECs from both genotypes exhibited minimal expression of these cytokines (Fig. 6F, G and Supplementary Fig. 5C, D). Efforts to block the activities of these cytokines with neutralizing antibodies against IFNAR1, IL-6 and TNFα both in vivo and ex vivo failed to prevent premature thymic atrophy in DKO mice or the early collapse of DKO FTOCs when analyzed 18 days later with the regimens tested (data not shown). Given that mTECs express a broad array of cytokines that are absent or minimally expressed by cTECs (Supplementary Table 2) [17], identifying the specific cytokine(s) whose dysregulation drives altered TEC and thymocyte differentiation in the absence of Zfp36l1 and Zfp36l2 remains a significant challenge.

## Discussion

In the present study, we demonstrated that the embryonic and early neonatal thymuses of Zfp36l1/2 DKO mice maintained normal overall cellularity despite a reduction in the number of TECs during this period. This preservation is likely driven by elevated FOXN1 protein expression and enhanced induction of prolymphopoietic target genes, resulting from diminished ARE-mediated *Foxn1* mRNA degradation in the absence of Zfp36l1 and Zfp36l2. However, a clear difference in thymic cellularity between DKO and control mice emerged after the first postnatal week, temporally coinciding with a reversal in FOXN1 protein expression in DKO TECs, from initially elevated to subsequently reduced relative to that in controls. Thus, given that FOXN1 downregulation is recognized as a key driver of age-associated thymic involution, these findings strongly support the conclusion that reduced FOXN1 protein levels in DKO TECs contribute to the accelerated involution observed in these mice.

During the period of rapid postnatal thymic growth, the number of mTECs increases approximately 10-fold within the first week [22]. Concurrently, mTECs start to express IFN-β, a type I IFN, shortly after birth [31]. A recent study revealed an elevated type I IFN response profile in all thymic cell types examined by scRNA-seq, including TECs, fibroblasts, endothelial cells, dendritic cells, macrophages, and B cells, from postnatal days 7–10 [32]. While the exact timing of expression for other cytokines, including IL-6 and TNFα, has not yet been documented in the literature, it is likely to follow a similar postnatal induction pattern. In the context of a dysregulated increase in the per-cell production of proinflammatory cytokines following the loss of two major posttranscriptional negative regulators, it is likely that elevated nonphysiological levels of intrathymic inflammatory cytokines impose significant stress on both developing thymocytes and supporting stromal cells. The observed temporal correlation between inflammatory cytokine accumulation and a decrease in FOXN1 expression suggests that increased cytokine production by DKO mTECs may suppress *Foxn1* transcription and override the reduced degradation of *Foxn1* mRNA caused by the deletion of these two RNA-binding proteins. In support of this hypothesis, bioinformatics analysis of scRNA-seq data from human fetal TECs revealed that *FOXN1* is potentially regulated by IRF1 and IRF8, two transcription factors activated by interferon signaling [33]. Nonetheless, a direct mechanistic link between inflammatory signaling and FOXN1 repression remains to be established.

However, blocking the activity of type I IFNs, IL-6 and TNFα concurrently was not sufficient to prevent early thymic involution. Neonatal mTECs produce a plethora of cytokines, both proinflammatory and anti-inflammatory, whereas both mTECs and cTECs express the receptors for these cytokines [17]. It is highly plausible that some other proinflammatory cytokines also contribute to premature thymic involution in the dual absence of Zfp36l1 and Zfp36l2.

Mutations in the *AIRE* gene lead to autoimmune polyendocrinopathy-candidiasis-ectodermal dystrophy (APECED), a systemic autoimmune disorder [4, 5]. High-titer neutralizing autoantibodies against type I IFNs are frequently found in APECED patients with *AIRE* mutations [34] and are associated with poor clinical outcomes in patients with COVID-19 and other viral infectious diseases [35, 36, 37, 38, 39]. Additionally, autoantibodies targeting IL-6 have been detected in *AIRE*-deficient patients [40]. These findings underscore the importance of endogenous expression of these proinflammatory cytokines by mTECs to establish central tolerance to these cytokines and ensure effective antiviral immunity. Furthermore, a recent study demonstrated that type I IFNs contribute to central tolerance induction beyond simply acting as tolerogenic self-antigens presented by thymic antigen-presenting cells [31]. This study revealed that IFN expression by mTECs actively promotes the activation and maturation of multiple types of antigen-presenting cells and facilitates the selection of regulatory T cells. Collectively, these studies highlight the indispensable role of mTEC-derived proinflammatory cytokines, particularly type I IFNs, in the induction of central tolerance. The results of the present study extend these insights by showing that cytokine expression must be tightly regulated to prevent premature thymic involution and identifying Zfp36l1 and Zfp36l2 as key posttranscriptional regulators that maintain this delicate balance.

## Materials and methods

### Mice

All mice were bred and maintained under specific pathogen-free conditions in the Preclinical Research Facility at Sunnybrook Research Institute. All animal procedures were approved by the Sunnybrook Research Institute Animal Care Committee and conducted in accordance with the committee’s ethical standards. The Zfp36l1 and Zfp36l2 double-floxed strain was generously provided by Dr. Martin Turner [21], and the Foxn1-Cre strain was obtained from The Jackson Laboratory [41]. Breeding these two strains resulted in Zfp36l1 and Zfp36l2 TEC-conditional double knockout mice, hereinafter referred to as DKO mice. The R26Foxn1 strain [42], a kind gift from Dr. Clare Blackburn, was crossed with Foxn1-Cre to produce the iFoxn1 strain, which expresses ectopic Foxn1 under the control of the CAG promoter from the Rosa26 locus in TECs.

### Cell preparation and flow cytometry analysis

For the TEC analysis, the embryonic thymus was incubated with 0.05% trypsin/EDTA at 37 °C for 15–30 minutes, depending on the age of the embryo, while postnatal thymic tissue was minced into ~1 mm pieces before digestion with a mixture of papain (0.5 mg/mL), collagenase IV (0.25 mg/mL), and DNase I (0.1 mg/mL) at 37 °C for 30–45 minutes [43]. Released cells were collected every 15 minutes, with fresh enzyme mixtures added each time. CD45^+^ hematopoietic cells were depleted using CD45 MicroBeads and LS columns (Miltenyi Biotec). When necessary, CD45^–^ stromal cells were stained with an APC-conjugated anti-EpCAM antibody (clone G8.8) and purified by fluorescence-activated cell sorting (FACS).

For DC and B-cell analysis, thymic tissues were minced into ~1 mm pieces and digested with collagenase IV (2.5 mg/mL) and DNase I (1 mg/mL) at 37 °C for 30 min. The majority of thymocytes were depleted using CD4 and CD8 microbeads and LS columns before staining with antibodies against DC and B-cell markers. For thymocyte subtype, selection and maturation analysis, the thymocytes were mechanically dissociated by gentle passaging through 40 μm strainers to avoid proteolytic antigen degradation. RTOCs and FTOCs were dissociated using TrypLE/EDTA to minimize surface antigen loss.

For intracellular cytokine staining (TNFα and IL-6), thymic stromal cells (after CD45^+^ MACS depletion) were cultured with monensin (BD GolgiStop) for 4 h at 37 °C. The cells were then stained for viability and surface markers (CD45, EpCAM, Ly51, and UEA1), fixed and permeabilized using a BD Cytofix/CytoPerm™ solution kit, and stained intracellularly with PE-Cy7-conjugated anti-TNFα and APC-conjugated anti-IL-6 antibodies.

The complete list of antibodies used for flow cytometry analysis in this study is shown in Supplementary Table 3.

### Single-cell RNA sequencing (scRNA-seq) analysis

scRNA-seq was performed on sorted TECs using the 10x Genomics Chromium Next GEM Single-Cell 3’ Kit v3.1 (Cat# 1000269). In brief, CD45^–^EpCAM^+^ TECs from three DKO and three control 6-day-old pups were independently labeled using TotalSeq™-A anti-mouse hashtag antibodies (BioLegend). Equal numbers of cells from each sample were pooled prior to encapsulation to minimize batch effects, resulting in the recovery of ~10,000 cells. An additive hashtag oligo (HTO) primer (5’-GTGACTGGAGTTCAGACGTGTGCTCTTCCGATCT) was included in the cDNA amplification to enable efficient hashtag library preparation. Following cDNA cleanup, an HTO-specific library was generated by amplification with primers targeting Nextera Read 1 and Read 2. Libraries were pooled and sequenced using paired-end 2 × 150 bp reads, with a 10% PhiX spike-in, targeting a depth of 40,000 reads per cell for the cDNA library and 500 reads per cell for the HTO library.

The demultiplexed sequencing data were analyzed using CellRanger v8.0.0 with default parameters, and the reads were aligned to the mouse genome and transcriptome (GRCm39-2024-A). Hashtag sequencing data were analyzed using CITE-seq-Count v1.4.5. The resulting count matrices were merged using Seurat v5.2.1, with doublet removal and hashtag call using the HTODemux function. Data normalization was performed using SCTransform v0.4.1, followed by principal component analysis (PCA), uniform manifold approximation and projection (UMAP), and clustering via the Louvain algorithm. Cell populations were annotated on the basis of gene signatures from a previous study [44]. Pathway enrichment analysis was performed using ReactomeGSA v1.18.0.

### Reaggregate thymic organ culture (RTOC)

Single-cell suspensions of E15.5 fetal thymuses were prepared by enzymatic digestion with 0.05% trypsin/EDTA at 37 °C for 15 min. A subset (5%) of dissociated cells was used for genotyping with Platinum Direct PCR Universal Master Mix. The remaining cells were pooled by genotype, and CD45^-^ stromal cells were isolated via MACS depletion. For the RTOC setup, 300,000 CD45^+^ hematopoietic cells and 300,000 CD45^-^ stromal cells were combined per aggregate. The cultures were analyzed after one and three weeks.

### EdU in vivo labeling and flow cytometric analysis

Timed-pregnant females were intraperitoneally injected with 2 mg of EdU, and embryos were collected at specified timepoints postinjection: 1 h (E13.5), 2 h (E15.5), or 4 h (E18.5). Single-cell suspensions of embryonic thymus were prepared enzymatically, stained for CD45 and EpCAM, fixed, permeabilized, and stained for incorporated EdU using the Click-iT™ Plus EdU Alexa Fluor 488 Flow Cytometry Kit.

### Bone marrow engraftment

CD3-depleted bone marrow cells from either CD45.1 wild-type C57BL/6J mice or CD45.2 *Ifnar1* knockout mice (a kind gift from Dr. David Brooks, University of Toronto) were intravenously injected into 4-week-old lethally irradiated recipients (10.5 Gy total body γ-irradiation, delivered 4 h before transplantation). Thymic reconstitution was assessed five weeks post-transplantation.

### Statistical analysis

Unpaired *t* tests (Holm–Šídák method) were performed for all statistical analyses unless specified otherwise in the figure legends using GraphPad Prism version 10.4.1 for macOS. ns indicates *P* > 0.05, whereas *, ***, and **** indicate *P* ≤ 0.05, 0.01, 0.001, and 0.0001, respectively.

### Supplementary material

The gating strategies for flow cytometry analysis are shown in Fig. S1. Observations supporting that Zfp36l1 plays a dominant role over Zfp36l2 in regulating the mTEC phenotype in 7-week-old mice are shown in Fig. S2. The results of the analysis related to the scRNA-seq data, including the proportions of individual clusters, RNA velocity and signaling pathway analysis, are shown in Fig. 3. EdU pulse-labeling results in embryonic TECs, the presence of an ARE within the *Foxn1* mRNA 3’UTR, and the similarity between DKO TECs and iFoxn1 TECs are shown in Fig. S4. The RTOC results, thymus cellularity in DKO mice engrafted with bone marrow cells from various sources, and splenic T-cell composition are shown in Fig. S5. Supplementary Table 1 lists the differentially expressed genes from our scRNA-seq experiment. Supplementary Table 2 lists the expression levels of selected cytokines in neonatal cTECs and mTECs according to publicly available data from St-Pierre et al. [17]. All the flow cytometry antibodies used in this study are listed in Supplementary Table 3.

## Supplementary information

Supplementary Table 1

Supplementary Table 2

Supplementary Table 3

Supplementary Figure 1

Supplementary Figure 2

Supplementary Figure 3

Supplementary Figure 4

Supplementary Figure 5

## Data Availability

The scRNA-Seq data, shown in Fig. 4 and Supplementary Fig. 3, are available in the GEO data repository (accession number: GSE315892).
