# Supplementary material for: RNA-binding proteins Zfp36l1 and Zfp36l2 protect against premature thymic involution
Source: Cell Mol Immunol. 2026 Mar 16;23(5):505–16. doi: 10.1038/s41423-026-01399-7 (PMC13129036; doi:10.1038/s41423-026-01399-7)
Supplement: Supplementary file 4 — Supplementary Figure 1 [file 41423_2026_1399_MOESM4_ESM.pdf]

Supplementary Figure 1

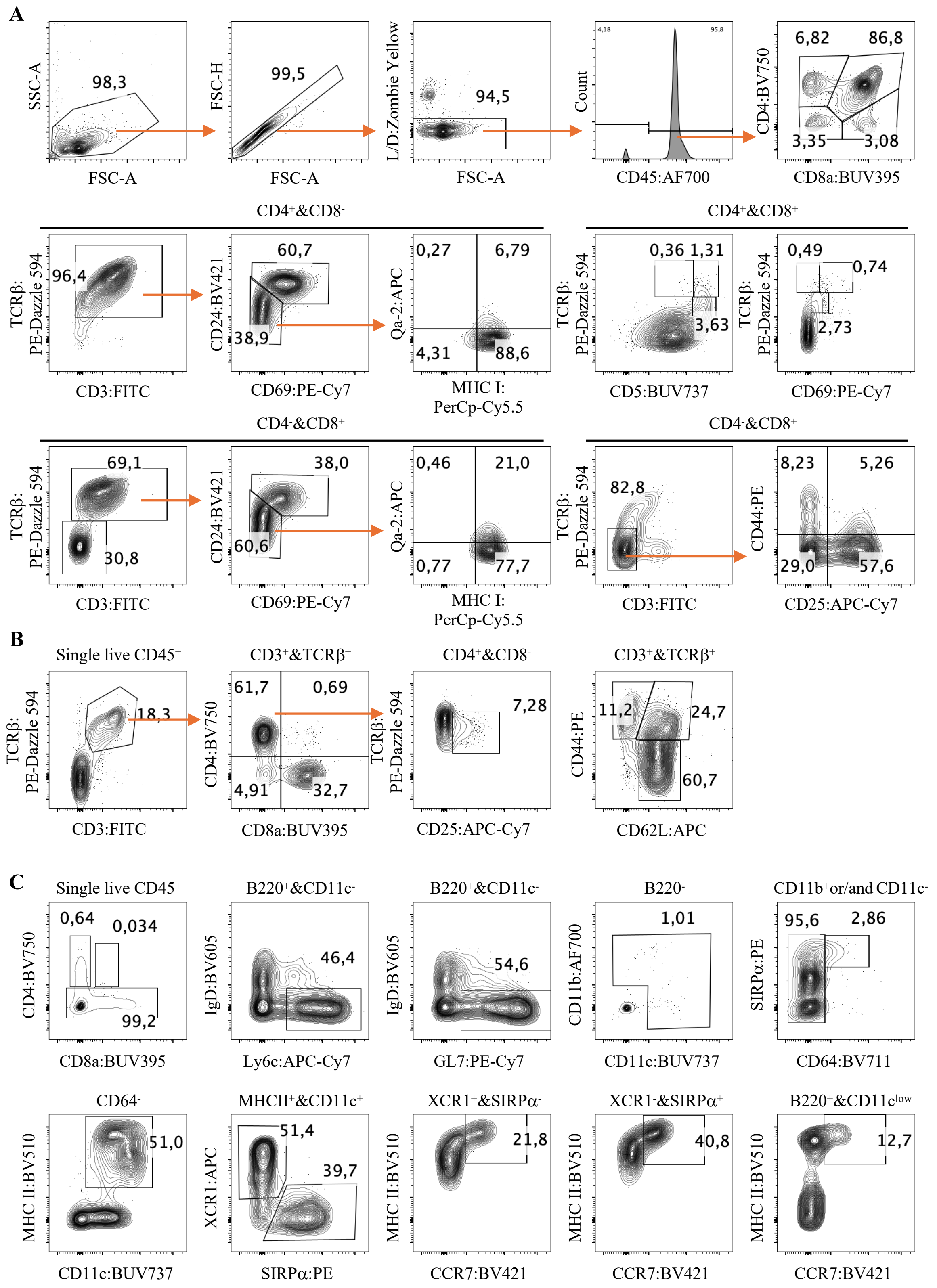

Supplementary Figure 1. Gating strategies for flow cytometry analysis. A) Gating strategies for thymocyte subsets, selection and maturation analysis. B) Gating strategies for spleen T cell analysis. The single live $CD45^{+}$ were the same as in A). C) Gating strategies for thymic antigen-presenting cell analysis as used in Ashby et al., 2024
