# Supplementary material for: RNA-binding proteins Zfp36l1 and Zfp36l2 protect against premature thymic involution
Source: Cell Mol Immunol. 2026 Mar 16;23(5):505–16. doi: 10.1038/s41423-026-01399-7 (PMC13129036; doi:10.1038/s41423-026-01399-7)
Supplement: Supplementary file 5 — Supplementary Figure 2 [file 41423_2026_1399_MOESM5_ESM.pdf]

Supplementary Figure 2

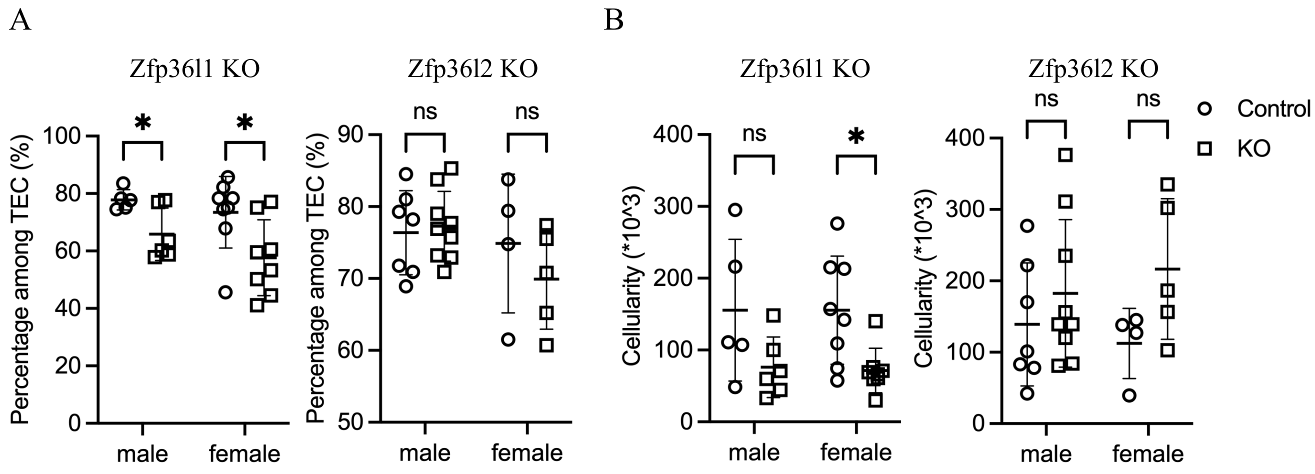

Supplementary Figure 2. Zfp36l1, but not Zfp36l2, deficiency in TECs leads to a significant reduction in Ly51-&UEA1+ mTECs in adult mice. A) Scatter plots comparing the percentage of mTEC among total TECs in control (○) and *Zfp36l* or *Zfp36l2* single-knockout (□) mice at 7 weeks of age. B) Scatter plots comparing the absolute number of mTEC between control (○) and *Zfp36l* or *Zfp36l2* single-knockout (□) mice at 7 weeks of age.
