# Supplementary material for: RNA-binding proteins Zfp36l1 and Zfp36l2 protect against premature thymic involution
Source: Cell Mol Immunol. 2026 Mar 16;23(5):505–16. doi: 10.1038/s41423-026-01399-7 (PMC13129036; doi:10.1038/s41423-026-01399-7)
Supplement: Supplementary file 6 — Supplementary Figure 3 [file 41423_2026_1399_MOESM6_ESM.pdf]

Supplementary Figure 3

A

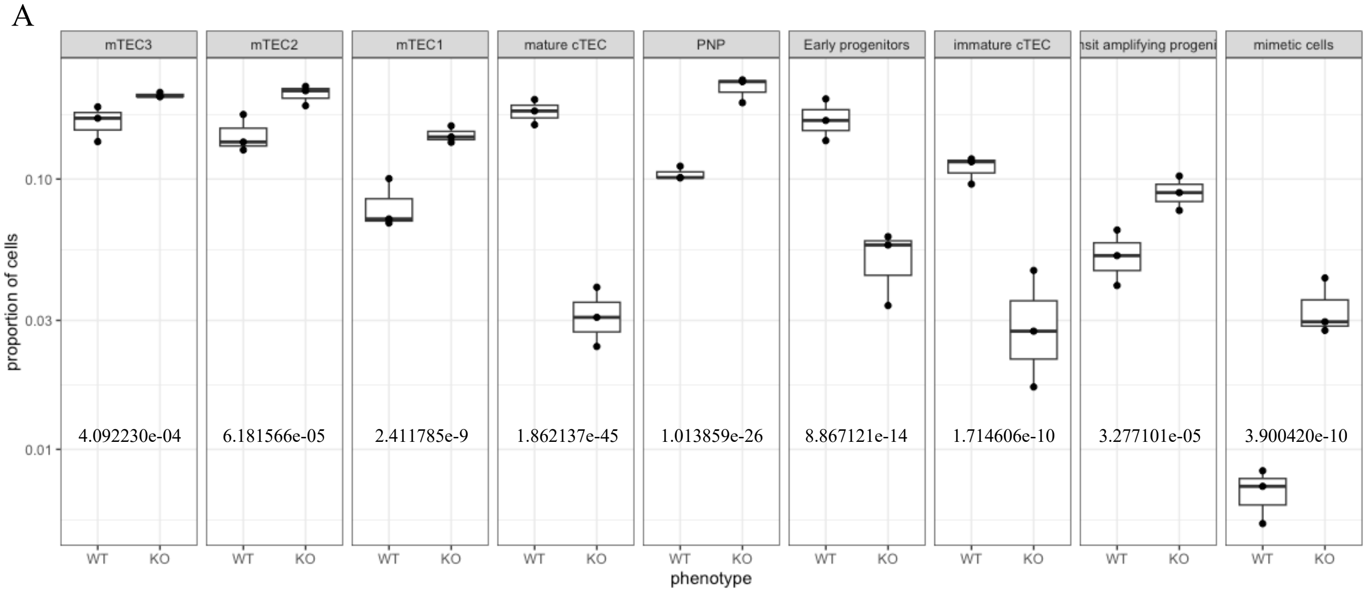

B

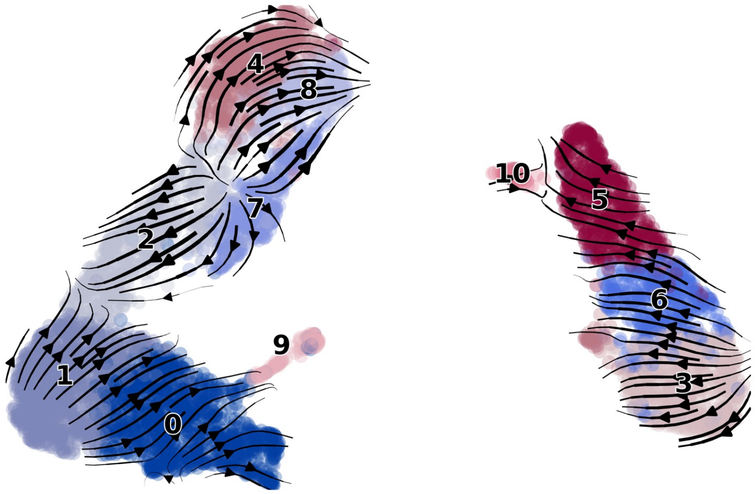

C

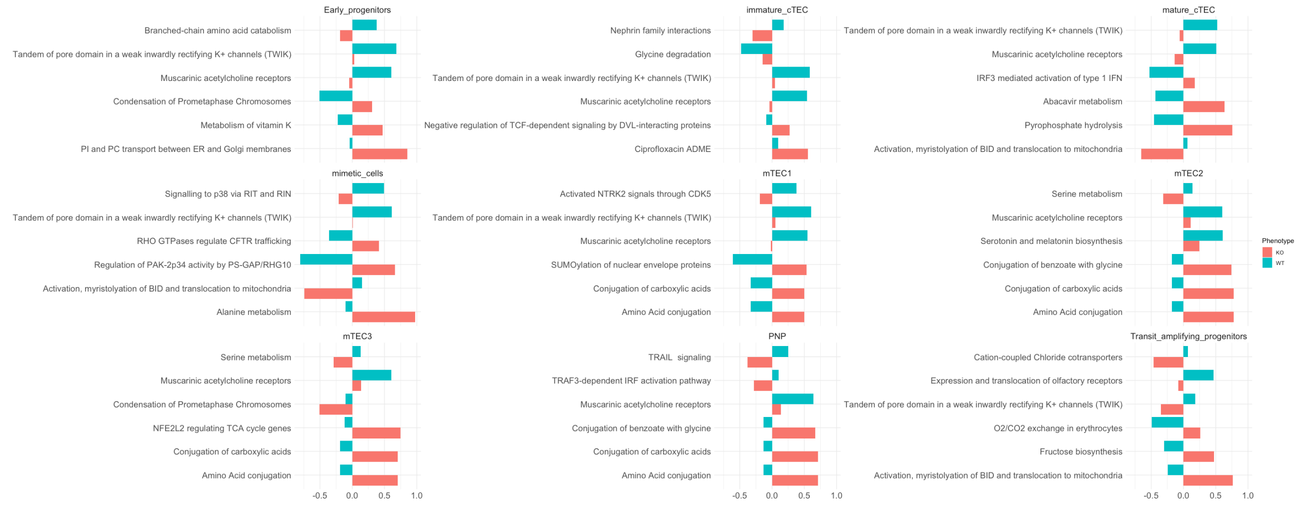

Supplementary Figure 3. A) A boxplot showing the proportions of each clusters in control and DKO TEC samples. The values inside the boxes are adjusted p values. B) RNA velocity analysis indicating potential differentiation trajectory. C) Signaling pathways affected by *Zfp36l1* and *Zfp36l2* deficiency in individual neonatal TEC subpopulations. Bar plots show enriched signaling pathways in differentially expressed genes from each TEC subpopulation of 6-day-old DKO versus control thymuses, as identified by gene set enrichment analysis.
