# Supplementary material for: RNA-binding proteins Zfp36l1 and Zfp36l2 protect against premature thymic involution
Source: Cell Mol Immunol. 2026 Mar 16;23(5):505–16. doi: 10.1038/s41423-026-01399-7 (PMC13129036; doi:10.1038/s41423-026-01399-7)
Supplement: Supplementary file 7 — Supplementary Figure 4 [file 41423_2026_1399_MOESM7_ESM.pdf]

Supplementary Figure 4

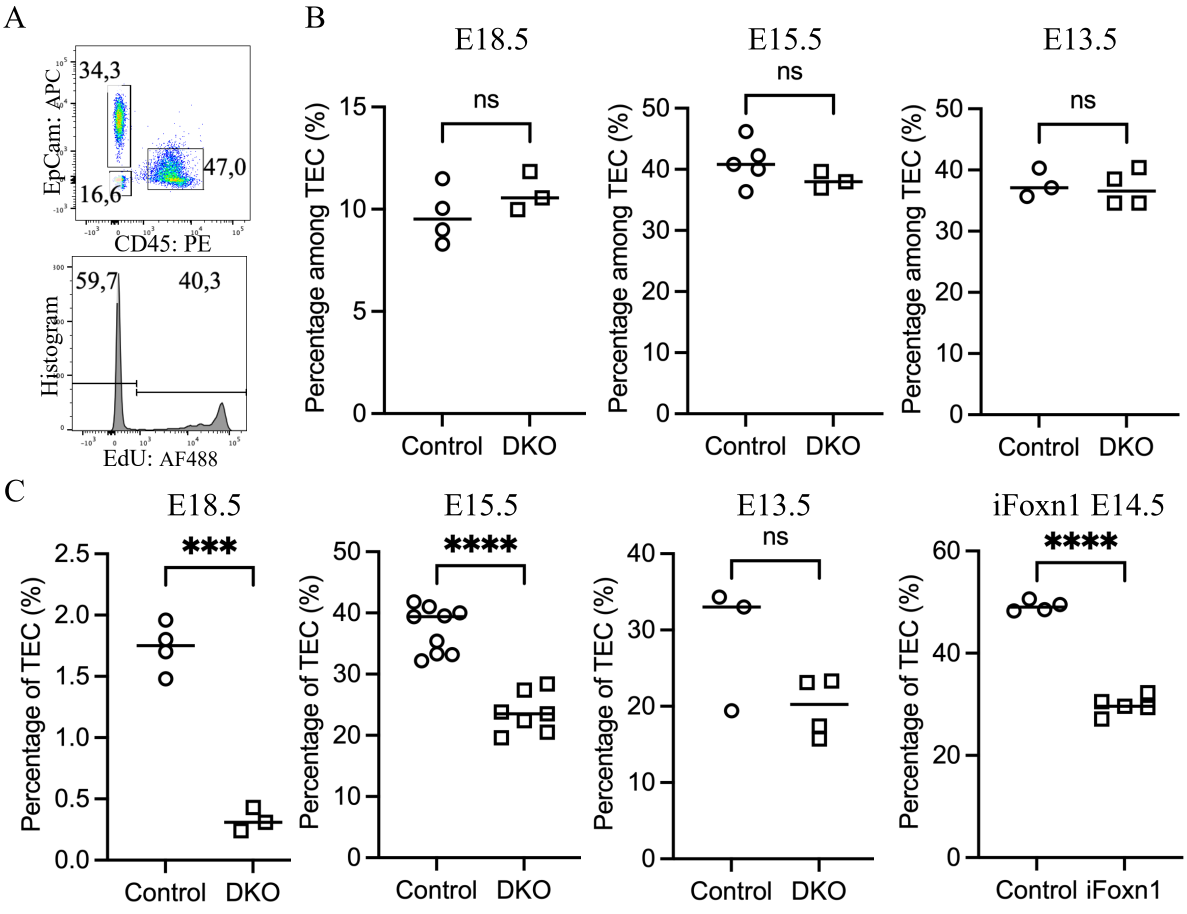

D

2561 UCUAUUUAUU CUCCCAUCUU ACACCCUUAA AGCCUACAGG GGCUCCUCCC
2601 GUAUGACCUG UGAACCCCUU AGGGAGACAA CUACUUCUAU GAAUUUAUCU

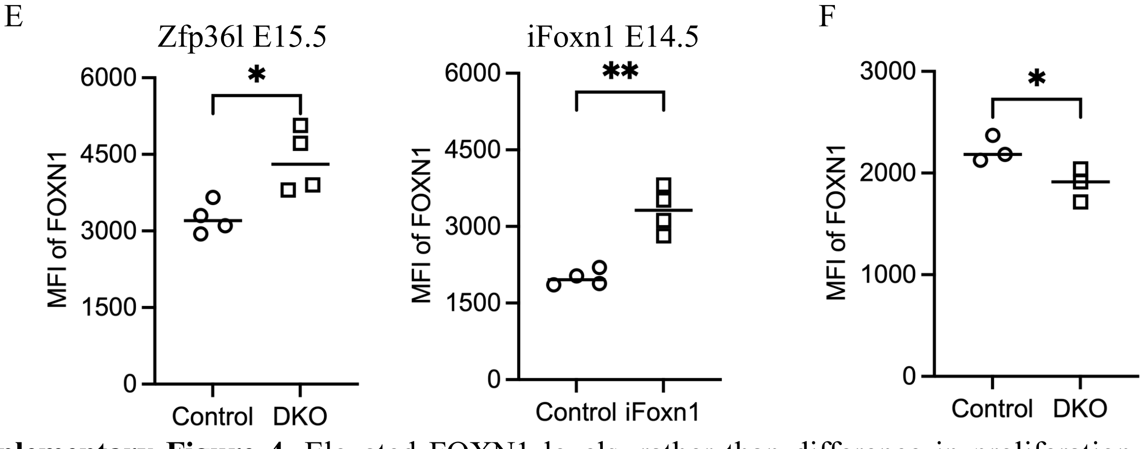

Supplementary Figure 4. Elevated FOXN1 levels, rather than difference in proliferation, account for reduced TEC frequency in embryonic DKO thymuses. A) Representative flow cytometry plots showing gating for CD45^{+} thymocyes, CD45^{-}EpCam^{+} TEC and CD45^{-}EpCam^{-} non-TEC stromal cells (top); EdU-labelled and unlabelled CD45^{-}EpCam^{+} TECs (bottom). B) Scatter plots comparing the percentages of EdU-labelled TECs between control (○) and DKO (□) thymuses from embryonic day (E) 13.5 (right), E15.5 (middle), and E18.5 (left). C) Scatter plots showing reduced TEC frequencies in DKO thymuses (□) at E13.5, E15.5, and E18.5 compared to floxed controls (○), resembling the phenotype observed in E14.5 iFoxn1 thymuses (□) compared to their respective controls (○). D) Partial sequence of the 3'UTR of *Foxn1* mRNA (NM_008238.2), highlighting the AU-rich elements in red. E) TECs from E15.5 *Zfp36l* DKO thymuses (□) express higher levels of FOXN1 protein than controls (○), a phenotype also observed in E14.5 iFoxn1 TECs (□) relative to their controls (○). F) A scatter plot comparing FOXN1 levels in TEC from 2-week control (○) and DKO (□) fetal thymus organ culture (FTOC).
