# Supplementary material for: RNA-binding proteins Zfp36l1 and Zfp36l2 protect against premature thymic involution
Source: Cell Mol Immunol. 2026 Mar 16;23(5):505–16. doi: 10.1038/s41423-026-01399-7 (PMC13129036; doi:10.1038/s41423-026-01399-7)
Supplement: Supplementary file 8 — Supplementary Figure 5 [file 41423_2026_1399_MOESM8_ESM.pdf]

Supplementary Figure 5

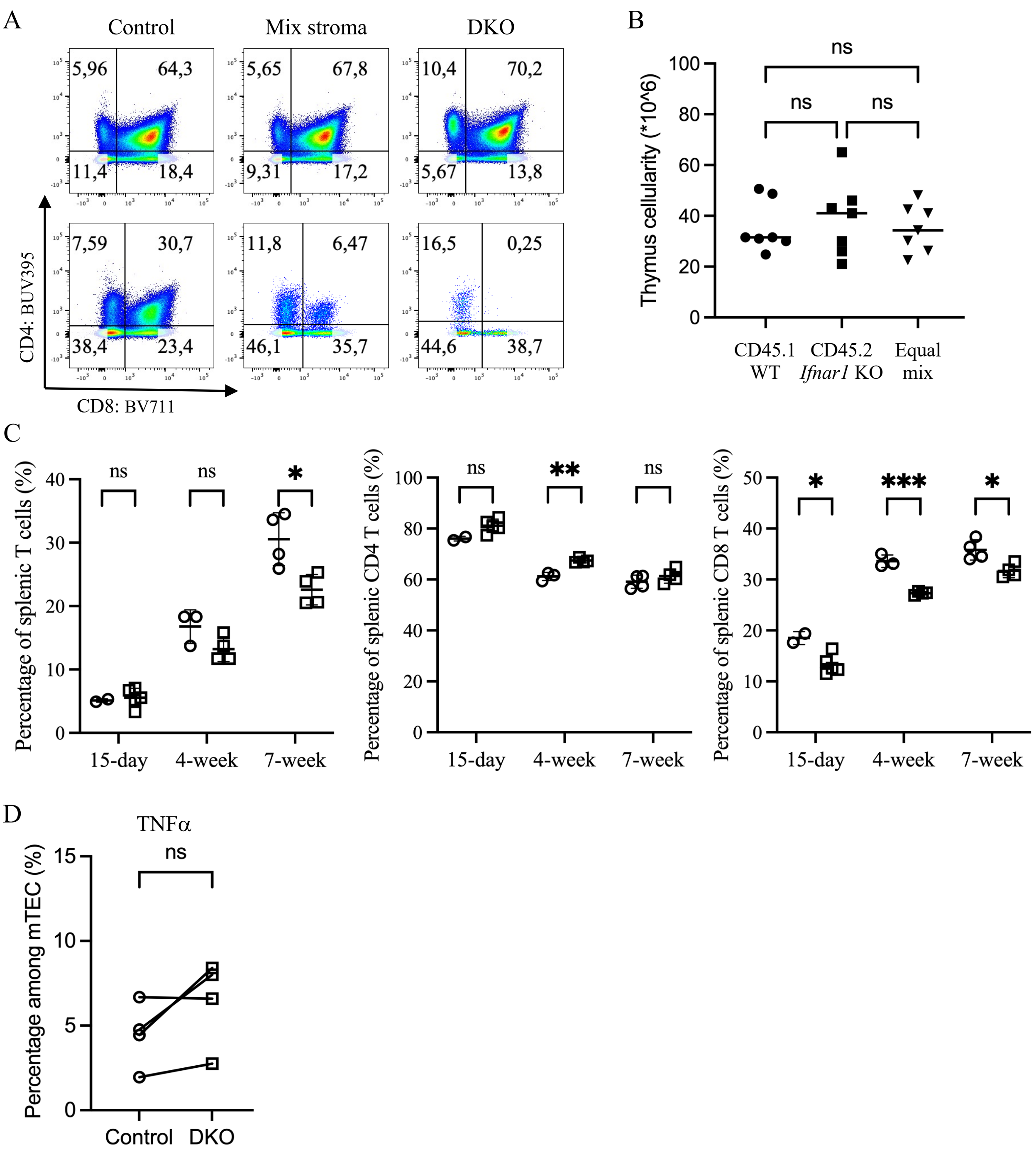

Supplementary Figure 5. The proinflammatory cytokine-mediated, cell-autonomous detrimental effect of DKO TECs on thymic size is not rescued by loss of responsiveness to type I IFNs in thymocytes. A) Representative flow cytometry contour plots of live CD45^{+} thymocytes from RTOC with stromal cells originated from E15.5 floxed control (left), DKO (right), or a 1:1 mixture of both (middle), showing comparable thymocyte development after 1-week (top), but an early loss of CD4^{+}CD8^{+} DP thymocytes in cultures containing DKO TECs after 3 weeks (bottom). B) A scatter plot comparing total thymus cellularity 5 weeks after transplantation of bone marrow from CD45.1 WT, CD45.2 *Ifnar1* KO, or a 1:1 mixture of both into whole-body lethally irradiated DKO host. One-way ANOVA analysis was used for statistical significance analysis. C) Scatter plots comparing the frequencies of T cell (left), and its CD4 (middle) and CD 8 (right) subsets, in spleens between control (○) and DKO (□) mice at 3 different ages. D) A scatter plot with paired *t*-test analysis comparing the percentages of IL6^{+} mTEC after 4-h GolgiStop treatment in control (○) and DKO (□) mice from postnatal day 9 to 3 weeks of age.
